# Genomic integration of lambda EG10 transgene in *gpt* delta transgenic rodents

**DOI:** 10.1186/s41021-015-0024-6

**Published:** 2015-12-01

**Authors:** Kenichi Masumura, Yasuteru Sakamoto, Wakako Kumita, Masamitsu Honma, Akiyoshi Nishikawa, Takehiko Nohmi

**Affiliations:** Division of Genetics and Mutagenesis, National Institute of Health Sciences, 1-18-1 Kamiyoga, Setagaya-ku, Tokyo 158-8501 Japan; Biological Safety Research Center, National Institute of Health Sciences, 1-18-1 Kamiyoga, Setagaya-ku, Tokyo 158-8501 Japan; Present address: Ajinomoto co., inc., Material Development & Application Labs, Research Institute For Bioscience Products & Fine Chemicals, 1-1 Suzuki-cho Kawasaki-ku, Kawasaki-shi, 210-8681 Kanagawa Japan

**Keywords:** *gpt* delta mouse and rat, Genomic rearrangement, Next generation sequencer, Copy number analysis

## Abstract

**Background:**

Transgenic *gpt* delta mouse and rat models were developed to perform *gpt* and Spi^−^ assays for *in vivo* mutagenicity tests. The animals were established by integration of lambda EG10 phage DNA as a transgene into the genome. The inserted position of the transgene on chromosome was determined by fluorescent *in situ* hybridization and Southern blot analyses; however, the exact position and sequence of the inserted junction were not known. To identify the site and pattern of genomic integration of the transgene copies, genomic DNAs extracted from C57BL/6J *gpt* delta mice and F344 *gpt* delta rats were applied to whole genome sequencing and mate-pair analysis.

**Results:**

The result confirmed that multi-copy lambda EG10 transgenes are inserted at a single position in the mouse chromosome 17. The junction contains 70 bp of overlapped genomic sequences, and it has short homology at both ends. A copy number analysis suggested that the inserted transgenes may contain 41 head-to-tail junctions and 16 junctions of other types such as rearranged abnormal junctions. It suggested that the number of intact copies could be approximately 40 at maximum. In the F344 *gpt* delta rats, transgenes are inserted at a single position in the rat chromosome 4. The junction contains no overlapped sequence but 72-kb genomic sequence including one gene was deleted. The inserted transgenes may contain 15 head-to-tail junctions and two rearranged junctions. It suggested that the number of intact copies could be 14 at maximum. One germline base substitution in the *gpt* gene rescued from *gpt* delta rats was characterized.

**Conclusions:**

The exact inserted positions of the lambda EG10 transgene in the genome of *gpt* delta transgenic rodents were identified. The copy number and arrangement of the transgene were analyzed. PCR primers for quick genotyping of *gpt* delta mice and rats have been designed.

**Electronic supplementary material:**

The online version of this article (doi:10.1186/s41021-015-0024-6) contains supplementary material, which is available to authorized users.

## Background

Transgenic rodent gene mutation assays are useful tools to detect *in vivo* mutagenicity in various types of rodent tissues [1]. These assays are based on transgenic animals that contain multiple copies of chromosomally integrated shuttle vectors that harbor reporter genes for the detection of gene mutations. The shuttle vector is recovered from genomic DNA of rodent tissues, and the mutated reporter genes can be phenotypically selected in a bacterial host cell. As a transgene, lambda phage DNA is used in some animal models [2–7]. The lambda phage shuttle vector is approximately 45–48 kb in size, and it is designed to contain reporter genes from lambda phage itself and those from *Escherichia coli*.

The transgenic mouse *gpt* delta was established via the microinjection of lambda EG10 phage shuttle vectors into the fertilized eggs of C57BL/6J mice [8]. Lambda EG10 was composed of lambda 2001 DNA [9] and a linearized plasmid flanked by two *loxP* sites. The plasmid region contains a replication origin, the chloramphenicol (Cm) resistance gene, and *gpt* of *E. coli* for 6-tihoguanine (6TG) selection to detect point mutations. The lambda region carries *red*, *gam*, and a *chiC* mutation for Spi^−^ selection to detect deletion mutations. Lambda EG10 DNA is 48 kb in size, and multiple copies of the transgene are integrated in a single position of the genome in a head-to-tail manner [8, 10, 11]. EG10 is located in chromosome 17B3 − C as detected by fluorescent in situ hybridization (FISH), and its copy number was estimated as approximately 80 per haploid by Southern blotting [8, 10]. The *gpt* delta rat was also established via microinjection of lambda EG10 DNA into the fertilized eggs of Sprague–Dawley (SD) rats [12]. Then, the F344 *gpt* delta rat was developed by backcrosses of the original SD *gpt* delta rat with wild type F344 rats [13]. The transgene is located in chromosome 4q24–31 as analyzed by FISH, and the copy number was estimated as approximately 10 per haploid by Southern blotting [12]. However, the exact position and sequence at the inserted junction were not identified in either the *gpt* delta mouse or rat. In other transgenic rodent models, Muta™Mouse carries approximately 40 copies of λgt10*lacZ* on chromosome 3 [14] and Big Blue^®^ mouse carries approximately 40 copies of λLIZα on chromosome 4 [2, 6]. Shwed et al. analyzed the copy number of transgenes in Muta™Mouse and Big Blue^®^ mouse by real-time PCR (RT-PCR) analysis [15]. They also reported some rearrangements of transgenes in the Muta™Mouse genome using a PCR-based genome scanning approach. The findings suggested the complexity of the genomic integration of transgenes in those rodent models.

In the past decade, next-generation sequencing (NGS) technology has been widely used in the field of genome science. To identify where and how the transgene is integrated in the genome of *gpt* delta transgenic rodents, genomic DNA extracted from male *gpt* delta mice and rats were applied to high-throughput DNA sequencing and mate-pair analysis by NGS. The exact sequences at the inserted junction of the transgene were identified. The copy number of the transgene was estimated by a non-PCR-based approach, and multiple rearrangements of the transgene were characterized. The result suggested that NGS has sufficient power to analyze complex rearrangements of multi-copy inserts in the genome.

## Methods

### High-throughput DNA sequencing analysis

**Fig. 1:**
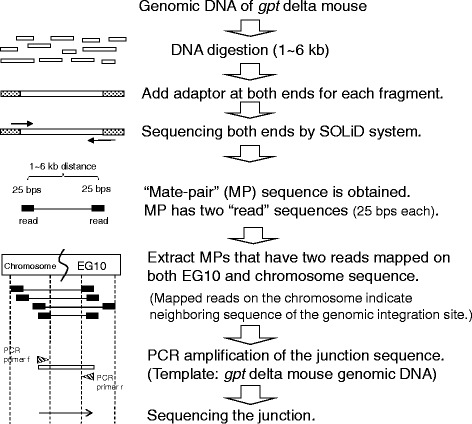
A brief protocol of identification of the insertion site of lambda EG10 in mouse chromosome

C57BL/6J *gpt* delta mice (transgene homozygous) and F344 *gpt* delta rats (transgene heterozygous) were obtained from Japan SLC (Shizuoka, Japan). The animal treatment of this study was approved by the Animal Care and Utilization Committee of the institute. Genomic DNA was extracted from the liver of one adult male *gpt* delta mouse or rat using a Wako DNA Extractor WB Kit (Osaka, Japan). Whole genome sequencing analyses were performed using the SOLiD System (Applied Biosystems by Life Technologies, Carlsbad, CA) by Dragon Genomics Center, TaKaRa BIO Inc. (Shiga, Japan). A brief protocol is shown in Fig. 1. The genomic DNA was digested using a Genemachines HydroShear (Digilab Genomic Solutions Inc., Holliston, MA) to produce a fragment distribution centered on 3 kb. Then, the fragments were used for emulsion PCR and high-throughput DNA sequencing (pair-end, 50 bps x 2). The DNA fragment with the sequenced pair-ends is named mate-pair (MP). MP is 1 ~ 6 kb in size and has two short sequences (reads) at the ends. For each MP, the sequences of the first 25 bases of each end were applied to data analysis as the reads. Sequence data were analyzed using the SOLiD System Color Space Mapping Tool (mapreads), Analysis Pipeline Tool (Corona), and Alignment Browser (SAB). The reference genome sequences used in the analyses include the C57BL/6J mouse genome, NCBI Build 37 mm9 [16], the F344 rat genome, UCSC rat genome rn4 [17], and the lambda EG10 transgene, lambda EG10 tentative sequence (48,416 bp) [18].

### Detection of the inserted position of the transgene in the genome

Sequenced reads were mapped on the reference sequences. First, the MPs for which one end was mapped on the lambda EG10 sequence were extracted. Next, the other end of the MPs was mapped on the mouse or rat genome sequence. The reads uniquely mapped on the reference sequence without mismatch bases were scored. Mapping information revealed the neighbor region of junctions (upstream and downstream) between the transgene and genome sequence. Based on the results of mapping, the appropriate PCR primers for both sides of the junction were designed. PCR was performed at 98°C for 3 min, followed by 25 cycles of 25 s at 98°C and 10 min at 68°C on a DNA Engine PTC-200 (MJ Research by Bio-Rad, Hercules, CA). PCR products were checked by agarose gel electrophoresis and sequenced using an ABI PRISM 3100 Genetic Analyzer (Applied Biosystems).

- Mouse upstream junction: forward (5′-GTTGTACTTCCAACCATGCCAAAG-3′) and reverse (5′-GTTCATCTGCTTTATGGGCAAGAG-3′)
- Mouse downstream junction: forward (5′-TGACTCGCTGCGCTCGGTC-3′) and reverse (5′-CAGAAATCATTCCAGGTCCTTGC-′)
- Rat upstream junction: forward (5′-GGTAGTGCCTCTTGTCCAGC-3′) and reverse (5′-ATTGCAGGCGCTTTCGCACTC-3′)
- Rat downstream junction: forward (5′-AACCGGGCTGGAAGCCGAG-3′) and reverse (5′-CCGTGTGGACCCTAGTCTAG-3′)

### Analyses of transgene integration and copy number

The copy number of transgenes integrated into mouse or rat genomes was estimated as follows. The transgene is integrated at a single site of the genome as multiple copies [1, 8, 10]. Therefore, there are two unique sites connecting transgene and genome sequences (upstream and downstream). The number of MPs for which one read was mapped on the transgene and the other read was mapped on the genome sequence indicated the number of MPs covering those unique inserted positions. The number of MPs covering two unique sites was divided by two to estimate the average number of MPs covering a single unique site in the genome. On the contrary, the number of MPs for which two reads were mapped on both the left and right arms of the transgene, indicating that the MPs covered the junction of two transgene copies, was counted. Then, the number of MPs covering the junction of two transgene copies was divided by the number of MPs covering a single unique site in the genome. The calculated multiplicity indicates the number of junctions of two transgene copies. Copy number of the transgene was estimated from the number of junctions. In addition, the integration pattern of multiple copies of the EG10 sequence was analyzed using the mapping data. The position and direction (plus and minus) of each read of the MPs were mapped on the EG10 sequence and plotted on a graph.

### Genotyping of the *gpt* delta mouse and rat

According to the sequence at the junction of the transgene integrated into genomic DNA, PCR primers were set for genotyping of *gpt* delta mice and rats:

- Mouse F1 (5′-GTTGTACTTCCAACCATGCCAAAG-3′),
- Mouse R1 (5′-CAGAAATCATTCCAGGTCCTTGC-3′),
- Mouse R3 (5′-CCCAGGTAATGAATAATTGCCTCTTTG-3′),
- Rat F1 (5′-TAGGGAGGCAGAGGCAGACG-3′),
- Rat R1 (5′-CCAGCTATGACTGGCATGTTACC-3′),
- Rat R2 (5′-TGCGGTATGAGCCGGGTCAC-3′).

Mouse F1-R1 amplifies a 360-bp product by the wild-type C57BL/6J allele. Mouse F1-R3 amplifies a 500-bp product by the C57BL/6J *gpt* delta allele. Rat F1-R1 amplifies a 400-bp product by the wild-type F344 allele. Rat F1-R2 amplifies a 175-bp product by the F344 *gpt* delta allele. PCR was performed with Ex Taq (TaKaRa BIO Inc.) at 98°C for 3 min followed by 40 cycles of 25 s at 98°C and 2 min at 68°C. PCR products were checked by 2% (W/V) agarose gel electrophoresis.

### Spot test for phenotypic characterization of the *gpt* mutants rescued from *gpt* delta rats

Lambda EG10 was rescued from F344 *gpt* delta rat genomic DNA by *in vitro* packaging using Transpack Packaging Extract (Agilent technologies, Santa Clara, CA). Rescued phages were infected with *E. coli* YG6020. Colonies possessing the converted plasmid containing *gpt* were grown on M9 with 25 μM Cm agar plates for 3 days at 37°C. In total, 30 Cm^r^ clones were randomly picked and sequenced for *gpt* using an ABI3100 Genetic Analyzer. Two of the clones possessed a T to A transversion at position 299 in *gpt*. These two *gpt* mutants and two control clones without mutations in *gpt* were incubated at 37°C in LB with Cm overnight. The overnight culture was diluted to a concentration of 1 × 10^0^–1 × 10^8^ with LB medium and spotted onto M9 with Cm or M9 with Cm and 25 μM 6TG agar plates. The plates were incubated at 37°C for two days. As positive and negative controls, YG6020 clones harboring *gpt*^*−*^ plasmids with a G to A transition at 185 or a GGG to GG deletion at 416–418 and *gpt*^+^ plasmid with no mutation in *gpt*, respectively, were used.

## Results

### Transgene integration in the mouse genome

**Fig. 2:**
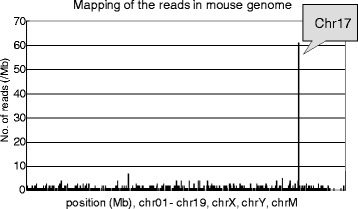
Insertion site of lambda EG10 in the mouse genome. Sequenced MPs carrying lambda EG10 and mouse chromosome sequences in each read were selected, and the mouse sequence reads were mapped on the reference mouse genome. The lambda EG10 transgene was integrated into chromosome 17

**Fig. 3:**
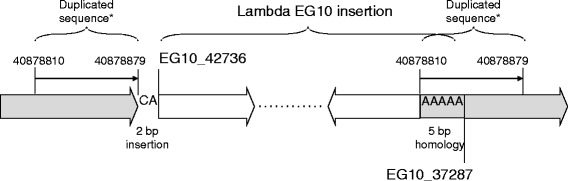
Sequence of the inserted junction of transgenes in *gpt* delta mouse genome. The white arrow represents the lambda EG10 transgene, and the dark arrow represents the mouse chromosome. Small arrows show the duplicated sequences in the mouse chromosome. *The short chromosome sequences are duplicated at the both junctions. (Chr17_40878810 to 40878879: 70 bps)

High-throughput DNA sequencing produced 3.1 × 10^8^ reads for the C57BL6/J male *gpt* delta mouse genome. The MPs for which one read was mapped on the lambda EG10 sequence were extracted. Then, the other reads of the MPs were mapped on the mouse genome sequence. Those reads were mapped at a single position in the upstream portion of chromosome 17 (Fig. 2). In other words, lambda EG10 transgenes are inserted at this position. The MPs were classified into upstream and downstream junctions. In the upstream junction, the MPs were mapped on the sequence of mouse chromosome 17 (40875881–40878795) at one end and the sequence of lambda EG10 (42775–46857) at the other end. In the downstream junction, the MPs were mapped on the sequence of EG10 (41458–37342) at one end and chromosome 17 (40878971–40882343) at the other end. For each junction, the remaining sequence gap between the transgene and mouse genome was less than 300 bp and filled by PCR and Sanger sequencing (Fig. 1). The sequence at the junction is shown in Fig. 3. At the junction, there was a 70-bp overlapped genome sequence (Chr17_40878810 to 40878879). There was a 2-bp insertion (+CA) upstream of the integration of EG10. Downstream of this region, there was a 5-bp overlapped sequence (AAAAA) between EG10 and the mouse chromosome. The direction of EG10 copies at both ends was inverted. Several kilobases of EG10 sequence ends were deleted at the upstream and downstream junctions. By integrating these transgenes, no disrupted gene was detected in the mouse genome.

**Fig. 4:**
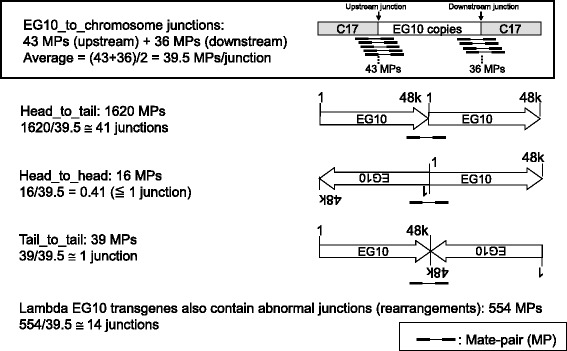
Number of junctions of lambda EG10 transgenes in *gpt* delta mice. The number of each junction was estimated by dividing the number of MPs with the average number of upstream and downstream MPs, i.e., 39.5

The copy number of EG10 was calculated by the number and proportion of the mapped MPs (Fig. 4). The numbers of MPs covering the integrated junction in the genome were 43 upstream and 36 downstream. Thus, the number of MPs covering a single unique site in the genome could be estimated as (43 + 36)/2 = 39.5. On the contrary, the number of MPs covering the junction between two transgene copies in a head-to-tail direction was 1620 MPs. Therefore, the number of head-to-tail junctions was estimated as 1620/39.5 = 41 junctions. This value may range 38–45, if it is calculated as 1620/43 or 1620/39. The numbers of MPs covering the transgenes in a head-to-head and tail-to-tail direction were 16 and 39 MPs, respectively. This suggests that there is at least one head-to-head junction and one tail-to-tail junction. The 16 MPs covering the head-to-head junction was less than 39.5 MPs as one junction. It may indicate that there is a small deletion at the head-to-head junction. Interestingly, another type of MP was observed as transgenes that are joined with the fragmented ends. These are abnormal junctions containing fragmentation or rearrangement of transgene copies. The number of MPs for the abnormal junctions was 554. Thus, the number of these abnormal junctions was estimated as 554/39.5 = 14 junctions.

Next, the integration pattern of multiple copies of the EG10 sequence was analyzed. The position and direction (plus or minus) of each read of MPs were mapped on the transgene and plotted on a 2D graph (Additional file 1: Fig. S1). The number of abnormal junctions was also counted as 14 based on the number of colonies of dots plotted on the graph (Additional file 2: Fig. S2). This value was consistent with the number calculated as described above.

**Fig. 5:**
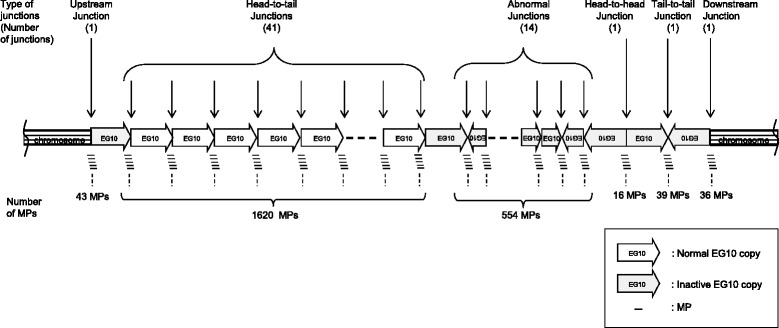
Schematic representation of genomic integration of the EG10 copies in *gpt* delta mouse. This figure represents a conceptual diagram of integration pattern of the EG10 copies in the genome. Sequential order of each EG10 copy is not identified. Stripe box at both ends represents mouse chromosome sequence. Thick arrows represent the EG10 copies and direction of the sequence. White arrows are intact EG10 copies. Dark arrows are rearranged inactive copies. Line arrows point the junctions between copies or between chromosome and EG10. Small lines represent the MPs covering the junctions. The calculated number of junctions is indicated in a parenthesis

These results suggest that the inserted transgenes may contain 41 head-to-tail junctions and 16 other type junctions (1 head-to-head, 1 tail-to-tail, and 14 fragmented junctions) per haploid genome (Fig. 5). The sequential order of those junctions was not identified. Then, the copy number of EG10 was calculated from the number of junctions (Additional file 3: Fig. S3). The number of intact copies of EG10 could be approximately 40 because fragmented copies or fusion copies cannot be rescued as lambda phages by lambda packaging reaction. Head-to-head and tail-to-tail junctions are also abnormal rearrangements because lambda DNA ends, namely a *cos* site with 12-bp cohesive termini (5′-G/GGGCGGCGACCT-3′), can ligate only in a head-to-tail manner as a functional multimer [19, 20]. PCR analysis could detect head-to-tail junctions of EG10 transgenes in the genomic DNA of *gpt* delta mice, but it failed to detect head-to-head and tail-to-tail junctions (Additional file 4: Fig. S4). This suggests that those junctions could be rearranged abnormal junctions. Those rearranged copies were estimated to be at least 18 in number (1 upstream, 1 downstream, 1 head-to-head, 1 tail-to-tail, and 14 rearranged junctions). The exact sequences of the junctions were not confirmed in this study. If some of head-to-tail junctions have small deletions, it may decrease the number of intact copies and increase the number of rearranged copies.

**Fig. 6:**
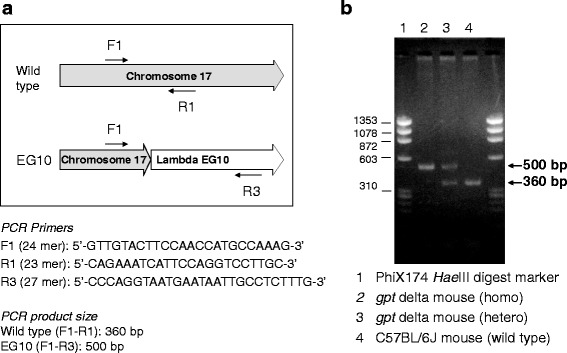
Genotyping of *gpt* delta mouse by PCR. Sequences and positions of PCR primers are presented (**a**). PCR condition is described in Materials and Methods. Image of agarose gel electrophoresis (**b**)

For quick genotyping of EG10-integrated *gpt* delta mice, PCR primers were designed (Fig. 6). Wild-type (F1-R1: 360 bp) and EG10 (F1-R3: 500 bp) loci were successfully detected by PCR with three primers.

### Transgene integration in the rat genome

**Fig. 7:**
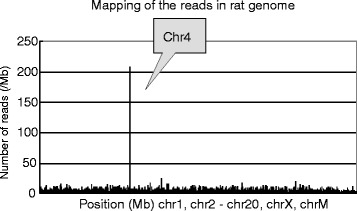
Insertion site of lambda EG10 in the rat genome. Sequenced MPs carrying lambda EG10 and rat chromosome sequences in each read were selected, and rat sequence reads were mapped on the reference rat genome. The lambda EG10 transgene was integrated into chromosome 4

**Fig. 8:**
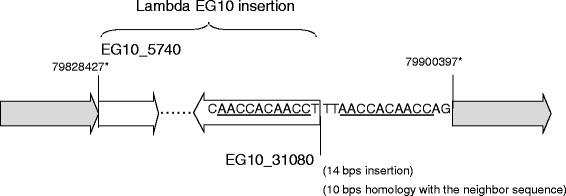
Sequence of the inserted junction of transgenes in the *gpt* delta rat genome. The white arrow represents the lambda EG10 transgene, and the dark arrow represents the rat chromosome. *The chromosome sequences are deleted at the junction. (Chr4_79828427 to 79900397: 71,789 bps)

High-throughput DNA sequencing produced 9.9 × 10^8^ reads for the male F344 *gpt* delta rat genome. The MPs for which one read was mapped on the lambda EG10 sequence were extracted, and the other reads of MPs were mapped on the rat genome sequence. The result indicated that the lambda EG10 transgene is inserted at a single position in chromosome 4 (Fig. 7). The MPs were classified into upstream and downstream junctions. In the upstream junction, the MPs were mapped on the sequence of rat chromosome 4 (79824561–79828298) at one end and the sequence of lambda EG10 (5739–9868) at the other end. In the downstream junction, the MPs were mapped on the sequence of EG10 (34542–31243) at one end and chromosome 4 (79900421–79903033) at the other end. For each junction, the remaining sequence gap between the transgene and rat genome was less than 200 bp and filled by PCR and Sanger sequencing. The sequence at the junction is shown in Fig. 8. The junction contained no overlapped genome sequence, but 72-kb rat sequence was deleted (Chr4_79828427 to 79900397). By loss of 72-kb sequences in the rat genome via integration of the transgenes, one gene, *Snx10* [RGD: 1305782] coding sorting nexin 10, was partially deleted. The upstream junction was a blunt-end junction between EG10 and the rat chromosome. At the downstream junction, there were 14-bp insertion sequences. In this insertion, there was homology with 10 bp in the neighbor EG10 sequence. The direction of EG10 copies at both ends was inverted. EG10 sequences were fragmented at the upstream and downstream junctions.

**Fig. 9:**
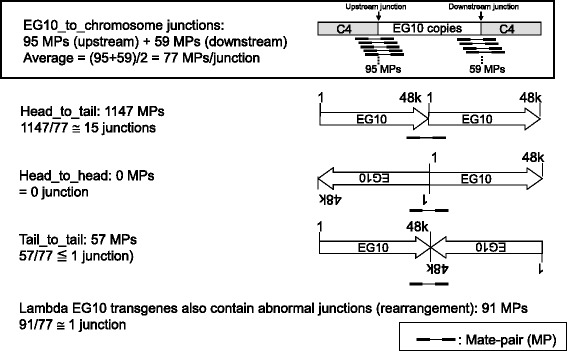
Number of junctions of lambda EG10 transgenes in *gpt* delta rats. The number of each junction was estimated by dividing the number of MPs with the average number of upstream and downstream MPs, i.e., 77

The copy number of EG10 was calculated as described above and is summarized in Fig. 9. The numbers of MPs covering the junction between the transgene and rat genome were 95 upstream and 59 downstream. Therefore, the number of MPs covering a single unique site in the genome could be estimated as (95 + 59)/2 = 77 MPs. The number of MPs covering the junction between two transgene copies in a head-to-tail direction was 1147. Thus, the number of head-to-tail junctions was estimated as 1147/77 = 15 junctions. This value may range 12–19, if it is calculated as 1147/95 or 1147/59. The numbers of MPs covering the transgenes in head-to-head and tail-to-tail directions were 0 and 57, respectively. This suggests that there are no head-to-head junctions and one tail-to-tail junction. The number of MPs for abnormal junctions containing rearrangement of transgene copies was 91. Therefore, the number of abnormal junctions was estimated as one. This junction was confirmed using the distribution map of MP dots (data not shown).

**Fig. 10:**
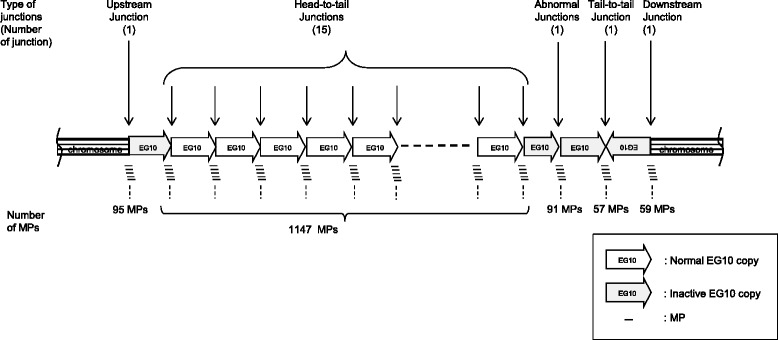
Schematic representation of genomic integration of the EG10 copies in *gpt* delta rat. This figure represents a conceptual diagram of integration pattern of the EG10 copies in the genome. Sequential order of each EG10 copy is not identified. Stripe box at both ends represents rat chromosome sequence. Thick arrows represent the EG10 copies and direction of the sequence. White arrows are intact EG10 copies. Dark arrows are rearranged inactive copies. Line arrows point the junctions between copies or between chromosome and EG10. Small lines represent the mate pairs (MPs) covering the junctions. The calculated number of junctions is indicated in a parenthesis

These results illustrate that the inserted transgenes may contain 15 head-to-tail junctions and two other type junctions (one tail-to-tail and one fragmented junction) per haploid genome (Fig. 10). PCR analysis could detect head-to-tail junctions in the genomic DNA of *gpt* delta rats (Additional file 4: Fig. S4). The number of intact copies of EG10 could be approximately 14. The number of rearranged or fragmented copies was estimated as at least four (one upstream, one downstream, and two rearranged junctions).

**Fig. 11:**
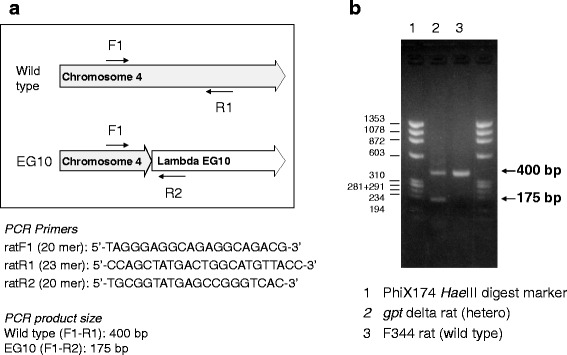
Genotyping of *gpt* delta rats by PCR. Sequences and positions of PCR primers are presented (**a**). PCR condition is described in Materials and Methods. Image of agarose gel electrophoresis (**b**)

PCR primers for the genotyping of *gpt* delta rats were designed (Fig. 11). Wild-type (F1-R1: 400 bp) and EG10 (F1-R2: 175 bp) loci were detected in *gpt* delta rats because these rats are maintained as heterozygotes.

### Characterization of a germline base substitution in *gpt* rescued from *gpt* delta rats

**Fig. 12:**
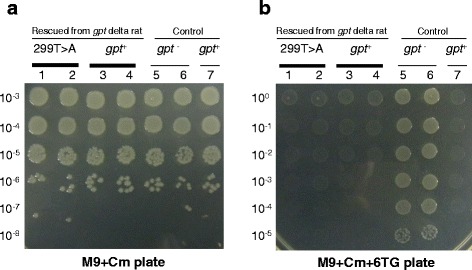
Characterization of the phenotype of the *gpt* mutants rescued from *gpt* delta rats by spot testing. The cultured *gpt* mutant clones were spotted onto M9 with Cm plates (**a**) and M9 with Cm and 6TG plates (**b**) in dilution series. The experimental condition was described in Materials and Methods. Lane 1 and 2: Clones which have the *gpt* mutation at 299 T to A. Lane 3 and 4: Clones which have no *gpt* mutation. Lane 5 and 6: Positive control which has a *gpt* mutation (185 G to A in lane 5 and 416–418 GGG to GG in lane 6). Lane 7: Negative control which has no *gpt* mutation. The A:T to T:A transversion at position 299 did not alter 6TG sensitivity as a negative control. Positive control *gpt* mutants exhibited 6TG resistance

In *gpt* mutation assays using *gpt* delta rats, we have frequently observed an A:T to T:A transversion at position 299 in the *gpt* sequence (aTt to aAt; Ile to Asn) (Additional file 5: Fig. S5) [21, 22]. The rescued *gpt* genes from F344 *gpt* delta rats without selection by 6TG were sequenced, and 2 of the 30 sequenced clones carried this mutation (data not shown). It is considered that the T to A mutation at position 299 does not cause the mutated *gpt* phenotype by itself. To confirm this finding, a spot test of *gpt* mutants was performed (Fig. 12). Overnight cultures of *E. coli* YG6020 clones carrying a plasmid containing *gpt* rescued from *gpt* delta rats were diluted and spotted onto agar plates containing Cm and 6TG. The clones containing *gpt* with the 299 T to A mutation were sensitive to 6TG similar to clones carrying wild-type *gpt*. On the contrary, positive control clones carrying *gpt* mutations such as G to A transitions or G deletions displayed a 6TG-resistant phenotype. These results indicate that the T to A mutation at 299 in *gpt* does not cause a 6TG-resistant phenotype and does not affect the mutation frequency in the assay.

## Discussion

In animal models developed for transgenic gene mutation assays, analysis of the genomic integration of multi-copy transgenes is not a simple issue. Transgenes are supposed to be randomly inserted at a single position of the host genome in a head-to-tail manner. In general, greater numbers of integrated lambda shuttle vectors in the host genome can result in higher rescue efficiency from the tissue sample. On the contrary, the megabase-size insertion of exogenous DNA into the genome may disrupt or delete host genes and affect the host animals. Therefore, characterization of the integrated transgene is important to understand what and how rearrangements occur with genomic integration. Chromosome analysis using FISH could identify the chromosomal position at which the transgene is inserted, but it could not identify the sequence at the inserted junction in detail. The signal intensity of Southern hybridization can provide a rough estimation of the copy number of transgenes. RT-PCR is another method to calculate the copy number of transgenes [15]. PCR-based approaches have a limitation for the analysis of high-copy-number or fragmented targets because the efficiency of amplification depends on specific primer sets.

In this study, we analyzed the genomic integration of lambda EG10 phage shuttle vectors in *gpt* delta mice and rats using NGS technology. In *gpt* delta mice, the MPs having lambda EG10 sequences in one end were mapped at a single position in the upper side of the chromosome 17B3 region (Figs. 2 and 3). It is consistent with the result obtained by FISH analysis [10]. It is notable that no genomic deletion was observed at the junction. It is consistent with the fact that we failed to search the integration site by comparative genomic hybridization (CGH) analysis (Mouse Genome CGH Microarray, Agilent Technologies) (data not shown). This reveals that NGS analysis could detect the inserted junction in such cases. On the contrary, the inserted EG10 sequences of several kilobases were partly deleted in both upstream and downstream junctions (Fig. 3). Using the identified sequence of the inserted junction, PCR primers were designed for quick genotyping of the *gpt* delta mouse (Fig. 6). The *gpt* delta mice have been used to investigate mutagenesis by crossing with other knockout and knockin mice such as *p53*, *Atm*, *Parp-1*, *Ogg1*, *Nrf2*, *Polk*, *IL-10*, and P450 reductase-null [23–34]. Quick typing of the transgene is useful to establish and maintain a specific genotype of a double transgenic mouse strains.

The copy number of the inserted transgene was estimated using a non-PCR-based method. The result indicated the presence of 41 normal junctions and 16 rearranged junctions (Figs. 4, 5). This suggests that the *gpt* delta mouse genome has approximately 40 EG10 copies in a head-to-tail manner and 18 rearranged copies per haploid. It was initially reported that the *gpt* delta mouse genome contains approximately 80 copies of the transgene per haploid as determined by Southern hybridization analysis [8]. In this study, the intact copy number was estimated as approximately 40 at maximum. Reasons for the different estimation include that Southern blot analysis is not necessarily quantitative and that many rearranged/fragmented copies are included in the integrated transgenes. Similar estimations are reported in the other models. The transgene copy number in Muta™Mouse was originally estimated as 35 per haploid by Southern hybridization and re-estimated as 29.0 ± 4.0 copies with approximately 10 defective fusion copies by RT-PCR [2, 15]. In the Big Blue^®^ mouse, it was estimated as 40 copies per haploid by Southern hybridization and as 23.5 ± 3.1 copies by RT-PCR [6, 15].

The integration pattern of multiple copies was analyzed by mapping of the MP reads on the lambda EG10 sequence (Additional file 1: Fig. S1). The 554 MPs were distributed into 14 rearranged junctions (Additional file 2: Fig. S2). Among them, seven junctions were arranged between two copies in the same direction, and the other seven junctions were fusions between reverse sequences. This is consistent with the result that the direction of EG10 copies at both genomic integration sites was inverted. All of these abnormal junctions resulted in deleted or fusion copies that could not be rescued by lambda packaging reaction because of size limitations or the lack of essential genes as a functional lambda phage particle [20, 35]. No hotspot of the rearrangement was observed. If the rearrangement occurred between multiple copies, the deleted or rearranged regions could be longer than 48 kb.

In the *gpt* delta rat, the analyses illustrated that the transgene is inserted at a single position in chromosome 4 (Fig. 7). There was a 72-kb deletion in the rat genomic sequence (Fig. 8). Because of the deletion, *Snx10* coding sorting nexin 10, was partially deleted. Interestingly, *gpt* delta rats that have lambda EG10 integration in both chromosome 4 alleles have not been established because of a defect of tooth development. It was reported that the gene function of *Snx10* may be involved in the regulation of endosome homeostasis [36]. Although the mechanism is not known and the gene-function relationship is not clear, it might be one candidate gene of the deficient phenotype. In addition to deletion of the coding sequence, insertion of mega-base exogenous DNA into the genome may also affect the gene expression in neighboring genomic regions.

In *gpt* delta rats, we have frequently observed an A:T to T:A transversion at position 299 in the *gpt* sequence [22]. The transgenes may contain inherited mutations in their sequences. If the original shuttle vector DNA had mutations, they would be maintained in the developed animals through the germline. Spontaneous germline mutations may also occur in animal breeding. It was reported that the EG10 sequence has some germline mutations such as base substitutions and indels [37]. In *gpt* assays, the A:T to T:A change at position 299 has been observed in untreated rats as well as mutagen-treated rats in different background (SD and F344) *gpt* delta rats and that mutation was also detected in the rescued lambda phages without 6TG selection. On the contrary, this mutation was never observed in *gpt* delta mice (among approximately 2,000 previously sequenced *gpt* mutants). In the *gpt* assay, this mutation was typically observed together with another *gpt* mutation in the same *gpt* mutants. Therefore, it is considered that the mutation itself does not cause the mutated *gpt* phenotype (6TG resistance). The bacterial spot test confirmed this hypothesis (Fig. 12), and therefore, the transversion at position 299 does not affect the *gpt* mutant frequency. Because of this evidence, the A:T to T:A transversion at 299 in *gpt* is an inherited mutation of *gpt* delta rats that should be excluded from the mutation spectra. Interestingly, it was previously reported that 20 % of *gpt* mutants recovered from *gpt* delta rats contain this base substitution and speculated that one of the five copies may have the A:T to T:A mutation at 299 [22]. Therefore, we concluded that this *gpt* mutation must have arisen in one lambda EG10 copy in the development of *gpt* delta rats. In this study, we revealed that two out of 30 rescued *gpt* genes had the A:T to T:A mutation. This frequency (1/15 = 7 %) is inconsistent with the frequency of 20 % described above. We speculate several possible reasons. First, some head-to-tail copies may contain point mutations and/or small deletions and those copies are functionally inactive in the rescue of transgene or mutation assay, and the workable copies may be approximately five. Second, although A:T to T:A mutation at 299 itself does not cause 6TG resistant phenotype, it may facilitate the mutated phenotype in combination with some types of the second mutation in *gpt*. In the phenotypic selection systems, there could be possible silent mutations in the reporter genes. They may change amino acid sequences of the gene products, but are not critical for enzymatic activities and do not result in phenotypic changes. The silent mutations depend on the sequence context and type of mutation. Further analysis using more number of the transgene rescued from *gpt* delta rats is required to understand the genetic property of each copy in detail.

Analysis of the transgene integration revealed many rearrangements of the transgene in the *gpt* delta rodent genome. When and how did these rearrangements occur? One possibility is that the rearrangements occurred in the early stage when the transgene is integrated into the host genome in the development of the transgenic animal. At that stage, exogenous lambda DNA is not methylated, and many DNA copies are injected into the fertilized egg. Although the mechanism of the genomic integration of exogenous DNA is not well known, the integrated transgenes may be unstable and cause complex rearrangements. Another possibility is that the genomic rearrangements may have occurred after the establishment of the animal line. If it occurs in the germline, the rearrangements may accumulate through animal breeding. Tandem copies of an approximately 50 kb sequence are concatenated at the single position in the host genome. Such regions may be good targets of genomic recombination. In fact, we have identified many complex rearrangements as spontaneous and mutagen-induced somatic Spi^−^ mutations in the tissues of *gpt* delta rodents [23, 28, 37–39]. However, a significant genotypic change such as loss of the transgene, change of the integration site in the genome, or pattern of genomic rearrangements was not reported, although many transgenic rodent models were developed more than 20 years ago [1]. In addition, the genotyping of *gpt* delta rodents using the PCR primers designed in this study works well among different generations. The transgenes are heavily methylated and not expressed in the host genome, suggesting that they are out of selection bias [2, 40]. Further work is required to clarify the timing and mechanisms underlying the complex genome rearrangements. The number and characteristics of the junctions or rearranged copies of transgenes could be a good signature to monitor the genomic rearrangements in future approaches. NGS technique could be useful to analyze not only transgenes but also genome-wide instability including complex genomic rearrangements.

## Conclusions

Genomic integration of the lambda EG10 transgene of *gpt* delta transgenic rodents was analyzed by whole genome sequencing and MP analysis. Copy number analyses estimated that the *gpt* delta mouse has approximately 40 head-to-tail copies with rearranged inactive copies and that the *gpt* delta rat has approximately14 head-to-tail copies with rearranged copies. The result suggested that NGS is a powerful tool for analyzing complex rearrangements of multi-copy inserts in the genome. Based on the sequence at the inserted junction, PCR primers were designed for quick genotyping of lambda EG10 alleles in *gpt* delta rodents.

## Additional files

Additional file 1: Fig. S1. Mapping of the mate pairs (MPs) covering the junctions of EG10 copies in *gpt* delta mice. Each MP has two sequenced positions (F and R reads). The sequenced reads were categorized as 4 types: F+, F position read EG10 sequence in plus direction; F−, F position read EG10 sequence in minus direction; R+, R position read EG10 sequence in plus direction; R−, R position read EG10 sequence in minus direction. The x and y axes represent the position in the EG10 sequence at which F and R reads was mapped, respectively. The white arrows represent the lambda EG10 copies, and the thick lines represent the sequenced reads of the MPs. EG10 DNA is approximately 48 kb in length, and the MPs are 1–6 kb in length. Each dot indicates an MP. For example, if the first read (F position) was mapped on the right end of lambda DNA and the other read (R position) was mapped on the left end and both reads are the same plus direction, that MP covers a head-to-tail junction (F + R+). Each junction is covered by MP dots in both plus and minus directions, and thus, the MP dots are symmetrically distributed with respect to a y = x line. On the graph, several types of junctions between EG10 copies are shown as colonies of the plotted dots: head-to-tail, head-to-head, tail-to-tail, and other abnormal junctions. (PPT 151 kb) Additional file 2: Fig. S2. Mapping of the abnormal junctions of EG10 copies in *gpt* delta mice. Abnormal junctions (554 mate-pairs) were mapped as spots shown as dotted circles. The x and y axes represent the position in the EG10 sequence at which F and R reads was mapped, respectively. Each junction has MPs in plus and minus directions, and thus, the spots are symmetrically distributed with respect to a y = x line. F + R+ spots are symmetrically paired with F − R− spots. F + R− and F − R+ spots are paired with the same type of spots. The paired spots represent c. In the upper side of y = x line of the graph, 14 spots are mapped as dotted circle. It indicates there are 14 junctions. Among them, seven junctions are arranged between two EG10 copies in the same direction (F + R+ and F − R−), and the other seven junctions are fusions between reverse sequences of EG10 (F + R− and F − R+). (PPT 109 kb) Additional file 3: Fig. S3. Estimation of the number of EG10 copies. (PPT 84 kb) Additional file 4: Fig. S4. PCR analysis of the type of junctions between lambda EG10 copies. PCR primers were set at both ends of lambda EG10 to amplify the connecting region of two EG10 copies. The genomic DNA of *gpt* delta mice and rats was used as the PCR template. PCR was performed as described in genotyping section in the Materials and Methods. (PPT 206 kb) Additional file 5: Fig. S5. The A:T to T:A transversion at position 299 in the *gpt* sequence. (PPT 102 kb)

## Abbreviations

6TG: 6-tihoguanine
CGH: Comparative genomic hybridization
Cm: Chloramphenicol
FISH: Fluorescent in situ hybridization
MP: Mate-pair
NGS: Next-generation sequencing
RT-PCR: Real-time PCR
SD: Sprague–Dawley
