# Supplementary material for: Genomic integration of lambda EG10 transgene in gpt delta transgenic rodents
Source: Genes Environ. 2015 Dec 1;37:24. doi: 10.1186/s41021-015-0024-6 (PMC4918054; doi:10.1186/s41021-015-0024-6)
Supplement: Additional file 1: Fig. S1. — Mapping of the mate pairs (MPs) covering the junctions of EG10 copies in gpt delta mice. Each MP has two sequenced positions (F and R reads). The sequenced reads were categorized as 4 types: F+, F position read EG10 sequence in plus direction; F−, F position read EG10 sequence in minus direction; R+, R position read EG10 sequence in plus direction; R−, R position read EG10 sequence in minus direction. The x and y axes represent the position in the EG10 sequence at which F and R reads was mapped, respectively. The white arrows represent the lambda EG10 copies, and the thick lines represent the sequenced reads of the MPs. EG10 DNA is approximately 48 kb in length, and the MPs are 1–6 kb in length. Each dot indicates an MP. For example, if the first read (F position) was mapped on the right end of lambda DNA and the other read (R position) was mapped on the left end and both reads are the same plus direction, that MP covers a head-to-tail junction (F + R+). Each junction is covered by MP dots in both plus and minus directions, and thus, the MP dots are symmetrically distributed with respect to a y = x line. On the graph, several types of junctions between EG10 copies are shown as colonies of the plotted dots: head-to-tail, head-to-head, tail-to-tail, and other abnormal junctions. (PPT 151 kb) [file 41021_2015_24_MOESM1_ESM.ppt]

## Slide 1
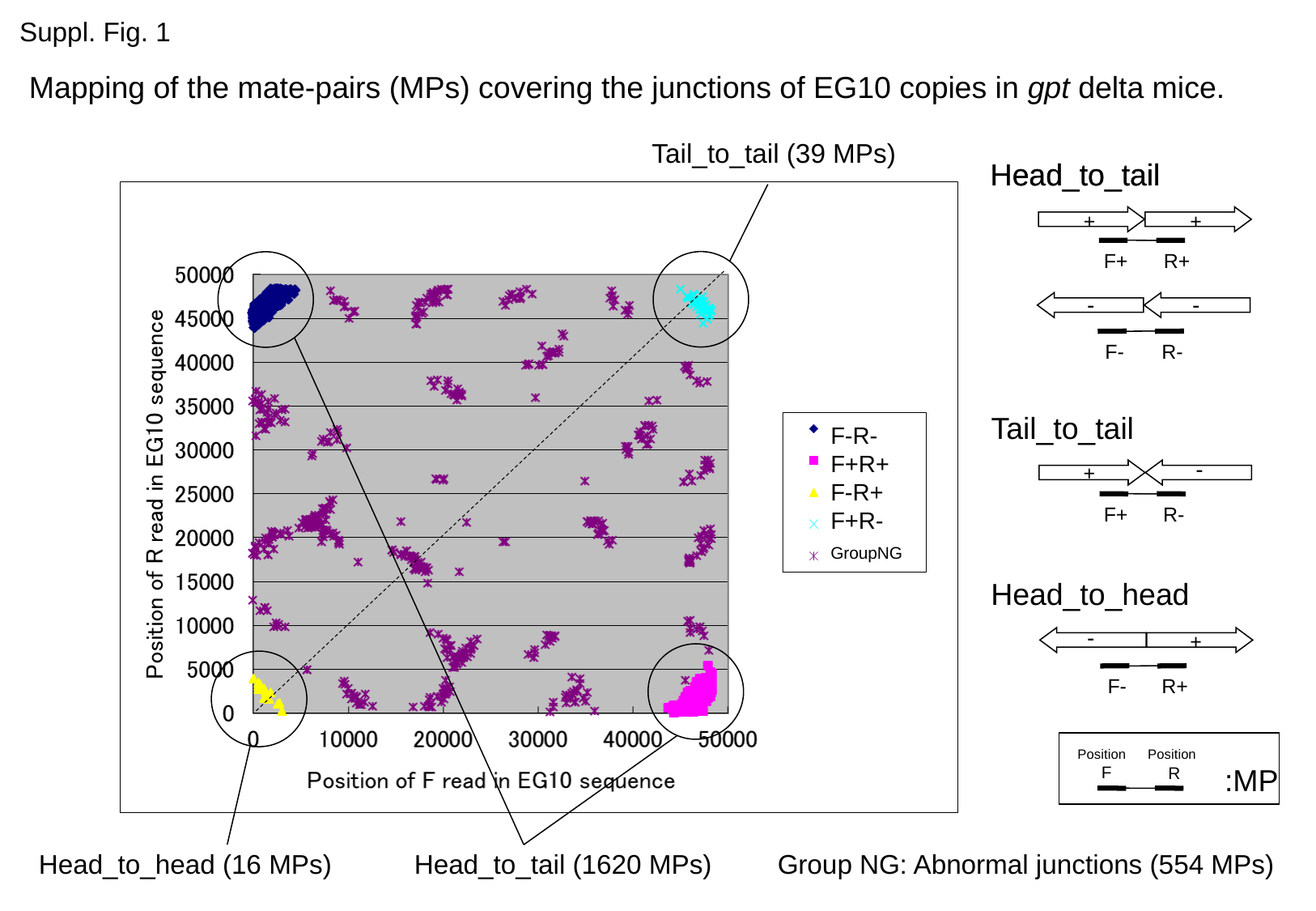

Suppl. Fig. 1
Mapping of the mate-pairs (MPs) covering the junctions of EG10 copies in gpt delta mice.
Tail_to_tail (39 MPs)
Head_to_tail
Head_to_tail
+
+
F+
R+
−
−
F-
R-
Tail_to_tail
F-R-
F+R+
F-R+
F+R-
GroupNG
−
+
F+
R-
Head_to_head
−
+
F-
R+
Position
R
Position
 F
:MP
Head_to_head (16 MPs)
Head_to_tail (1620 MPs)
Group NG: Abnormal junctions (554 MPs)
