# Supplementary material for: Genomic integration of lambda EG10 transgene in gpt delta transgenic rodents
Source: Genes Environ. 2015 Dec 1;37:24. doi: 10.1186/s41021-015-0024-6 (PMC4918054; doi:10.1186/s41021-015-0024-6)
Supplement: Additional file 2: Fig. S2. — Mapping of the abnormal junctions of EG10 copies in gpt delta mice. Abnormal junctions (554 mate-pairs) were mapped as spots shown as dotted circles. The x and y axes represent the position in the EG10 sequence at which F and R reads was mapped, respectively. Each junction has MPs in plus and minus directions, and thus, the spots are symmetrically distributed with respect to a y = x line. F + R+ spots are symmetrically paired with F − R− spots. F + R− and F − R+ spots are paired with the same type of spots. The paired spots represent c. In the upper side of y = x line of the graph, 14 spots are mapped as dotted circle. It indicates there are 14 junctions. Among them, seven junctions are arranged between two EG10 copies in the same direction (F + R+ and F − R−), and the other seven junctions are fusions between reverse sequences of EG10 (F + R− and F − R+). (PPT 109 kb) [file 41021_2015_24_MOESM2_ESM.ppt]

## Slide 1
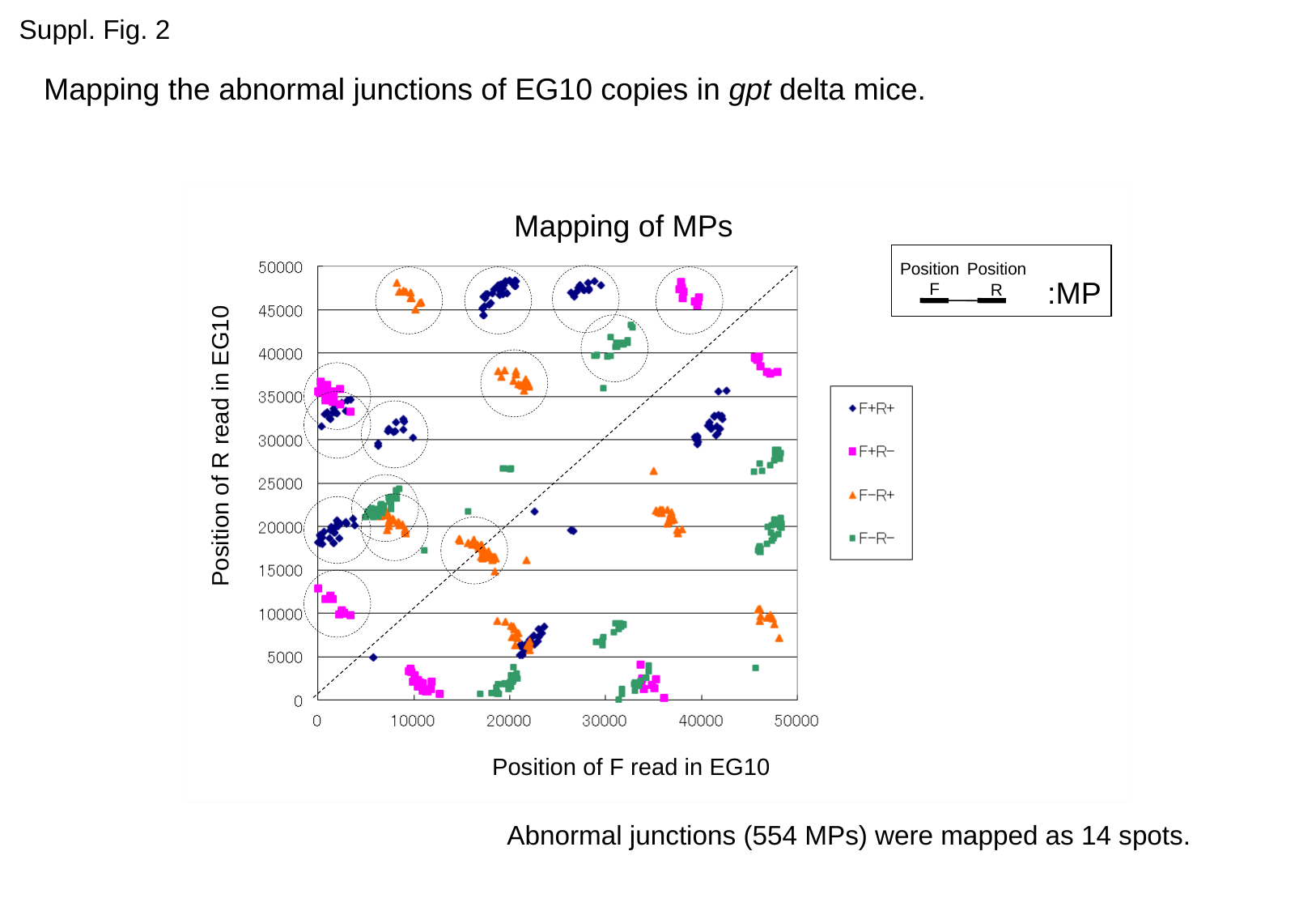

Suppl. Fig. 2
Mapping the abnormal junctions of EG10 copies in gpt delta mice.
Mapping of MPs
Position
 F
Position
R
:MP
Position of R read in EG10
Position of F read in EG10
Abnormal junctions (554 MPs) were mapped as 14 spots.
