# Supplementary material for: Genomic integration of lambda EG10 transgene in gpt delta transgenic rodents
Source: Genes Environ. 2015 Dec 1;37:24. doi: 10.1186/s41021-015-0024-6 (PMC4918054; doi:10.1186/s41021-015-0024-6)
Supplement: Additional file 3: Fig. S3. — Estimation of the number of EG10 copies. (PPT 84 kb) [file 41021_2015_24_MOESM3_ESM.ppt]

## Slide 1
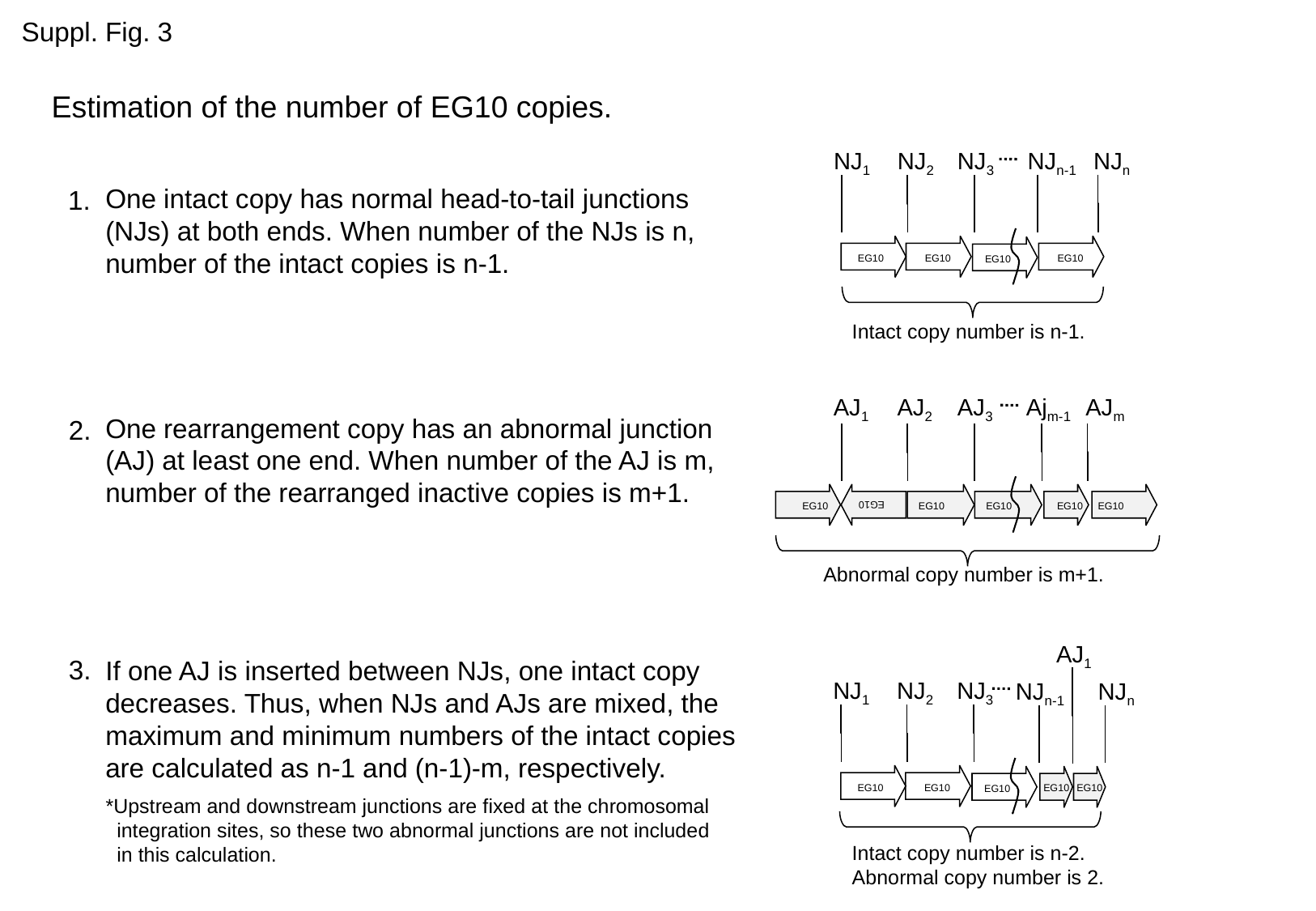

Suppl. Fig. 3
Estimation of the number of EG10 copies.
NJ1
NJ2
NJ3
NJn-1
NJn
One intact copy has normal head-to-tail junctions (NJs) at both ends. When number of the NJs is n, number of the intact copies is n-1.
1.
EG10
EG10
EG10
EG10
Intact copy number is n-1.
AJ1
AJ2
AJ3
Ajm-1
AJm
One rearrangement copy has an abnormal junction (AJ) at least one end. When number of the AJ is m, number of the rearranged inactive copies is m+1.
2.
EG10
EG10
EG10
EG10
EG10
EG10
Abnormal copy number is m+1.
AJ1
3.
If one AJ is inserted between NJs, one intact copy decreases. Thus, when NJs and AJs are mixed, the maximum and minimum numbers of the intact copies are calculated as n-1 and (n-1)-m, respectively.
NJ1
NJ2
NJ3
NJn-1
NJn
c
EG10
EG10
EG10
EG10
EG10
*Upstream and downstream junctions are fixed at the chromosomal integration sites, so these two abnormal junctions are not included in this calculation.
Intact copy number is n-2.
Abnormal copy number is 2.
