# Supplementary material for: Genomic integration of lambda EG10 transgene in gpt delta transgenic rodents
Source: Genes Environ. 2015 Dec 1;37:24. doi: 10.1186/s41021-015-0024-6 (PMC4918054; doi:10.1186/s41021-015-0024-6)
Supplement: Additional file 4: Fig. S4. — PCR analysis of the type of junctions between lambda EG10 copies. PCR primers were set at both ends of lambda EG10 to amplify the connecting region of two EG10 copies. The genomic DNA of gpt delta mice and rats was used as the PCR template. PCR was performed as described in genotyping section in the Materials and Methods. (PPT 206 kb) [file 41021_2015_24_MOESM4_ESM.ppt]

## Slide 1
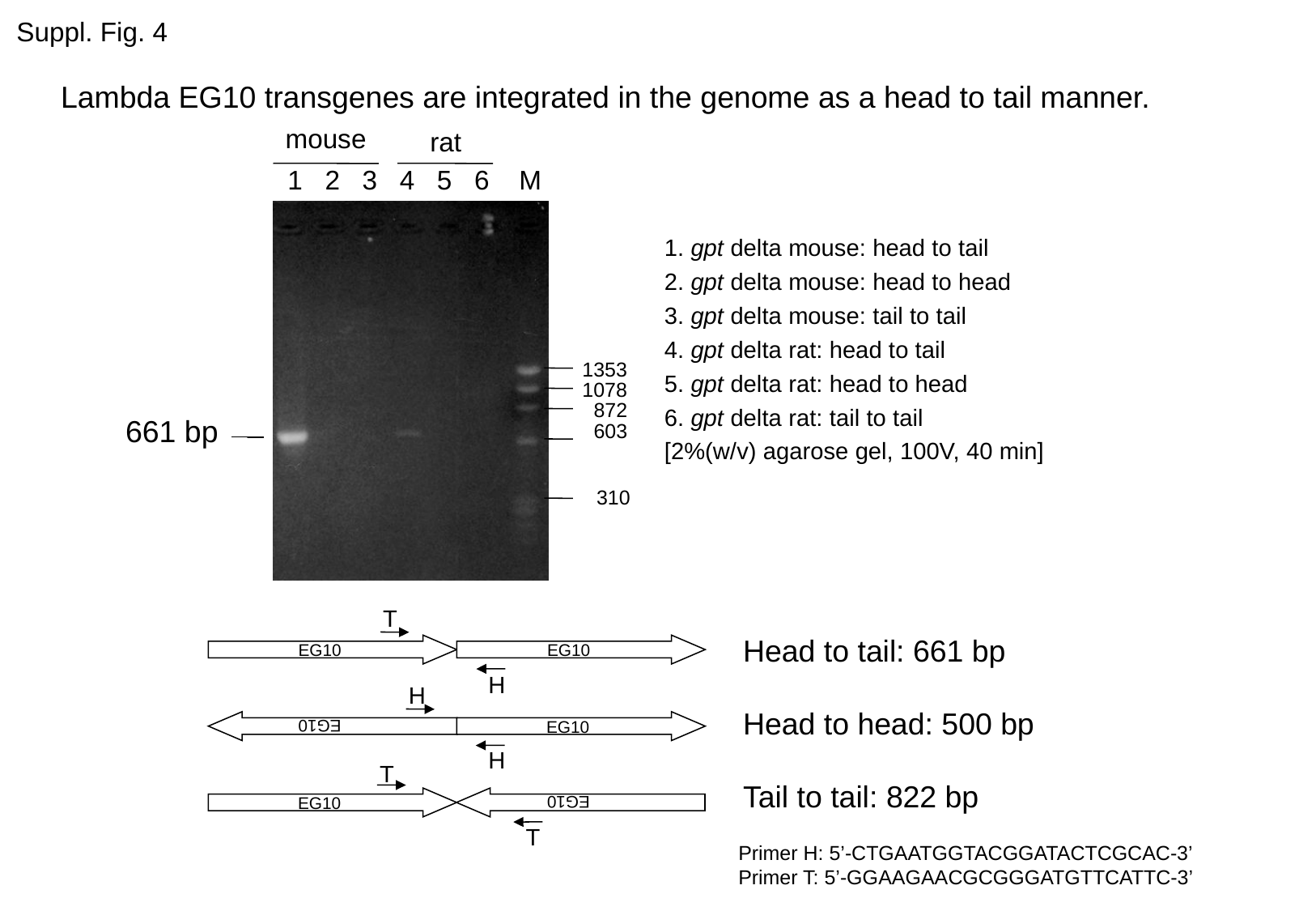

Suppl. Fig. 4
Lambda EG10 transgenes are integrated in the genome as a head to tail manner.
mouse
rat
1 2 3 4 5 6 M
1. gpt delta mouse: head to tail
2. gpt delta mouse: head to head
3. gpt delta mouse: tail to tail
4. gpt delta rat: head to tail
5. gpt delta rat: head to head
6. gpt delta rat: tail to tail
[2%(w/v) agarose gel, 100V, 40 min]
1353
1078
 872
661 bp
 603
 310
T
Head to tail: 661 bp
Head to head: 500 bp
Tail to tail: 822 bp
EG10
EG10
H
H
EG10
EG10
H
T
EG10
EG10
T
Primer H: 5’-CTGAATGGTACGGATACTCGCAC-3’
Primer T: 5’-GGAAGAACGCGGGATGTTCATTC-3’
