# Supplementary figures and images for: Genomic integration of lambda EG10 transgene in gpt delta transgenic rodents
Source: Genes Environ. 2015 Dec 1;37:24. doi: 10.1186/s41021-015-0024-6 (PMC4918054; doi:10.1186/s41021-015-0024-6)

## Slide 1
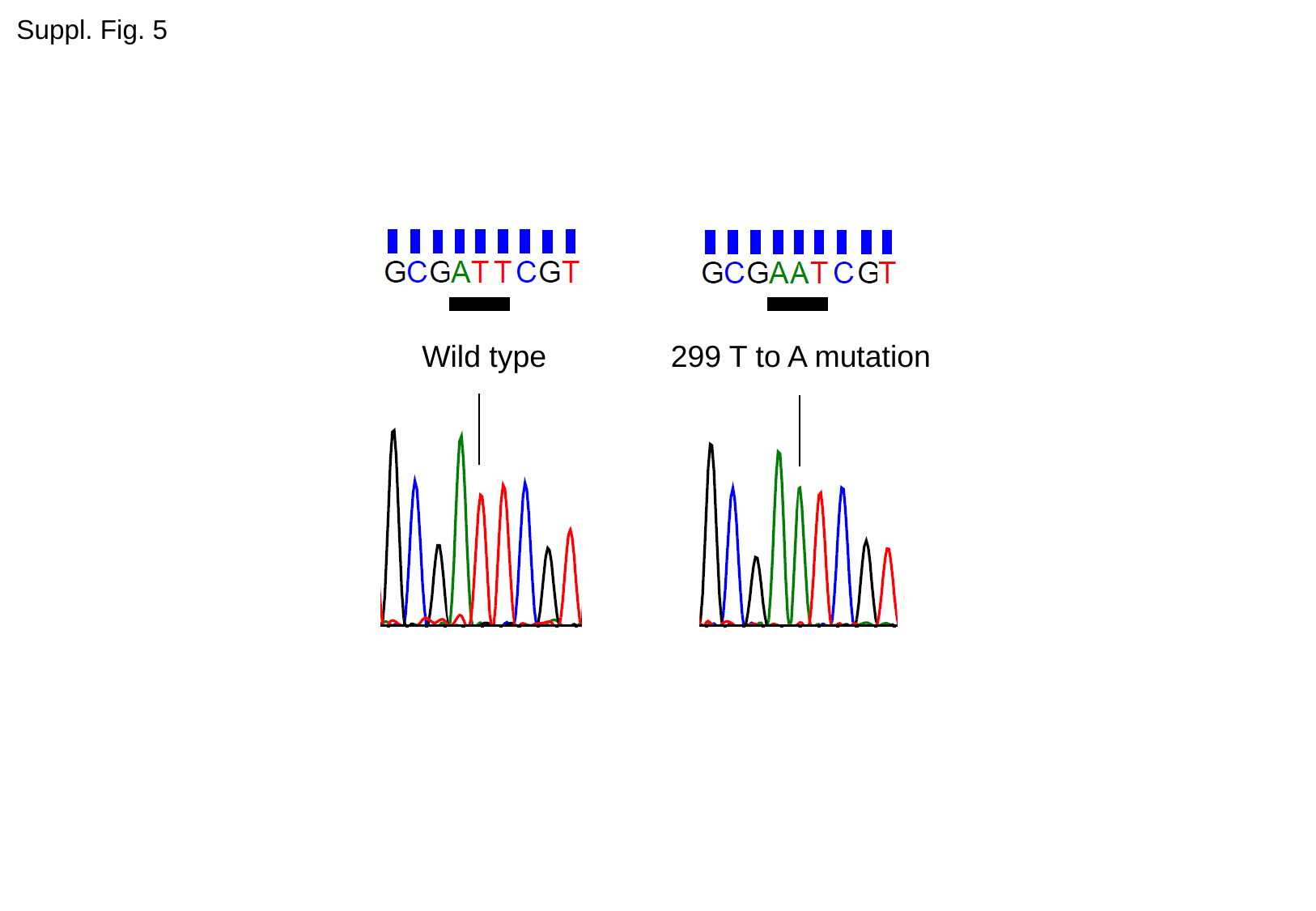

Suppl. Fig. 5
Wild type
299 T to A mutation

Supplement: Additional file 5: Fig. S5. — The A:T to T:A transversion at position 299 in the gpt sequence. (PPT 102 kb) [file 41021_2015_24_MOESM5_ESM.ppt]
